# Synthesis and Evaluation of the First ^68^Ga-Labeled *C*-Terminal Hydroxamate-Derived Gastrin-Releasing Peptide Receptor-Targeted Tracers for Cancer Imaging with Positron Emission Tomography

**DOI:** 10.3390/molecules29133102

**Published:** 2024-06-28

**Authors:** Lei Wang, Hsiou-Ting Kuo, Chao-Cheng Chen, Devon Chapple, Nadine Colpo, Pauline Ng, Wing Sum Lau, Shireen Jozi, François Bénard, Kuo-Shyan Lin

**Affiliations:** 1 Department of Molecular Oncology, BC Cancer Research Institute, Vancouver, BC V5Z 1L3, Canada; lewang@bccrc.ca (L.W.); htkuo0325@gmail.com (H.-T.K.); ccchen@bccrc.ca (C.-C.C.); devchapple@gmail.com (D.C.); ncolpo@bccrc.ca (N.C.); png@bccrc.ca (P.N.); wlau@bccrc.ca (W.S.L.); sjozi@bccrc.ca (S.J.); fbenard@bccrc.ca (F.B.); 2 Department of Molecular Imaging and Therapy, BC Cancer, Vancouver, BC V5Z 4E6, Canada; 3 Department of Radiology, University of British Columbia, Vancouver, BC V5Z 1M9, Canada

**Keywords:** gastrin-releasing peptide receptor, positron emission tomography, SB3, hydroxamate derivatives, gallium-68, pancreas uptake

## Abstract

Gastrin-releasing peptide receptor (GRPR), overexpressed in many solid tumors, is a promising imaging marker and therapeutic target. Most reported GRPR-targeted radioligands contain a *C*-terminal amide. Based on the reported potent antagonist D-Phe-Gln-Trp-Ala-Val-Gly-His-Leu-NHOH, we synthesized *C*-terminal hydroxamate-derived [^68^Ga]Ga-LW02075 ([^68^Ga]Ga-DOTA-pABzA-DIG-D-Phe-Gln-Trp-Ala-Val-Gly-His-Leu-NHOH) and [^68^Ga]Ga-LW02050 ([^68^Ga]Ga-DOTA-Pip-D-Phe-Gln-Trp-Ala-Val-Gly-His-Leu-NHOH), and compared them with the closely related and clinically validated [^68^Ga]Ga-SB3 ([^68^Ga]Ga-DOTA-pABzA-DIG-D-Phe-Gln-Trp-Ala-Val-Gly-His-Leu-NHEt). Binding affinities (K_i_) of Ga-SB3, Ga-LW02075, and Ga-LW02050 were 1.20 ± 0.31, 1.39 ± 0.54, and 8.53 ± 1.52 nM, respectively. Both Ga-LW02075 and Ga-LW02050 were confirmed to be GRPR antagonists by calcium release assay. Imaging studies showed that PC-3 prostate cancer tumor xenografts were clearly visualized at 1 h post injection by [^68^Ga]Ga-SB3 and [^68^Ga]Ga-LW02050 in PET images, but not by [^68^Ga]Ga-LW02075. Ex vivo biodistribution studies conducted at 1 h post injection showed that the tumor uptake of [^68^Ga]Ga-LW02050 was comparable to that of [^68^Ga]Ga-SB3 (5.38 ± 1.00 vs. 6.98 ± 1.36 %ID/g), followed by [^68^Ga]Ga-LW02075 (3.97 ± 1.71 %ID/g). [^68^Ga]Ga-SB3 had the highest pancreas uptake (37.3 ± 6.90 %ID/g) followed by [^68^Ga]Ga-LW02075 (17.8 ± 5.24 %ID/g), while the pancreas uptake of [^68^Ga]Ga-LW02050 was only 0.53 ± 0.11 %ID/g. Our data suggest that [^68^Ga]Ga-LW02050 is a promising PET tracer for detecting GRPR-expressing cancer lesions.

## 1. Introduction

Gastrin-releasing peptide receptor (GRPR) is a G protein-coupled receptor. It is expressed in some normal tissues and organs such as the pancreas, gastrointestinal tract, and nervous system, and regulates a series of physiological functions including stimulation of the contraction of smooth muscle cells and secretion of gastrointestinal hormones [1]. GRPR is also found overexpressed in several solid malignancies, such as prostate, breast, colon, and lung cancers, and is involved in the malignant neoplasm’s development by coupling with phospholipase C and activating protein kinase C [2,3,4,5,6,7]. Owing to its overexpression in malignant tissues, GRPR is a promising target for cancer imaging and therapy.

**Figure 1:**
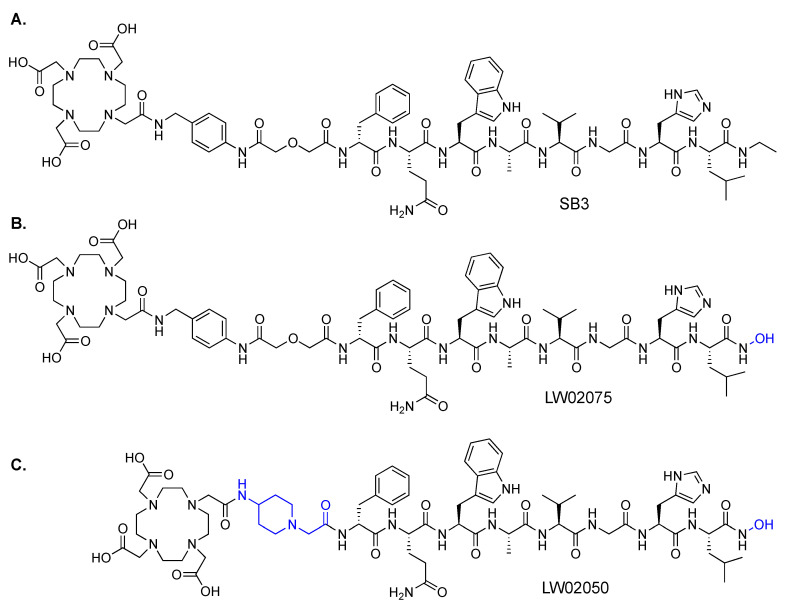
Chemical structures of (**A**) SB3, (**B**) LW02075, and (**C**) LW02050. The differences in the chemical structures between SB3 and the *C*-terminal hydroxamate-derived LW02075 and LW02050 are shown in blue.

In the past two decades, many radiolabeled GRPR-targeted ligands have been developed, and some of them have been translated into the clinic for cancer diagnosis and radioligand therapy [8,9,10,11,12]. [^68^Ga]Ga-SB3 (SB3: DOTA-pABzA-DIG-D-Phe-Gln-Trp-Ala-Val-Gly-His-Leu-NHEt; Figure 1A; pABzA-DIG: *p*-aminomethylaniline-diglycolic acid), developed by Maina et al., is one of the most popular radiolabeled GRPR antagonists, and has been validated in the clinic for detecting prostate and breast cancer lesions [13]. Although [^68^Ga]Ga-SB3 enabled clear visualization of prostate and breast cancer lesions in PET images, a very high pancreas uptake was observed in patients [13]. In addition to [^68^Ga]Ga-SB3, a high pancreas uptake is observed by other clinically validated GRPR-targeted radioligands including derivatives of AMBA, RM2 and NeoB [9,10,12]. The high uptake in pancreas not only affects lesion detection in or adjacent to the pancreas, but also significantly limits the maximum tolerated dose for targeted radioligand therapy application [1,13].

Most of the reported GRPR-targeted agonist and antagonist sequences contain a *C*-terminal amide. However, it seems that replacing the *C*-terminal amide with a *C*-terminal hydroxamate is tolerable, as a *C*-terminal hydroxamate-derived GRPR-targeted antagonist (D-Phe-Gln-Trp-Ala-Val-Gly-His-Leu-NHOH), reported by Devin et al., was shown to bind GRPR with a high affinity (K_i_ = 5.8 nM) [14]. To the best of our knowledge, no GRPR-targeted radioligands containing a *C*-terminal hydroxamate have ever been reported. Therefore, the goal of this study was to evaluate the potential of *C*-terminal hydroxamate-derived GRPR-targeted radioligands for cancer imaging, and document the extent of their uptake in the pancreas.

We used the chemical structure of [^68^Ga]Ga-SB3 (Figure 1A) as a template since it has been used to successfully detect prostate and breast cancer lesions in the clinic [13]. Additionally, the only difference between the GRPR-targeted sequence of [^68^Ga]Ga-SB3 (D-Phe-Gln-Trp-Ala-Val-Gly-His-Leu-NHEt) and the sequence reported by Devin et al. (D-Phe-Gln-Trp-Ala-Val-Gly-His-Leu-NHOH) is at the C-terminus. Therefore, we synthesized LW02075 (Figure 1B) by replacing the *C*-terminal *N*-ethylamide in SB3 with a *C*-terminal hydroxamate. Previously, our group reported two GRPR-targeted tracers, [^68^Ga]Ga-ProBOMB1 and [^68^Ga]Ga-ProBOMB2, based on the same GRPR-targeted sequence, [D-Phe^6^,Leu^13^ψPro^14^]Bombesin(6-14) [15,16]. We demonstrated that substitution of the pABzA-DIG linker in [^68^Ga]Ga-ProBOMB1 with a 4-amino-(1-carboxymethyl)piperidine (Pip) linker retained high tumor uptake but significantly reduced uptake in normal organs, particularly in the pancreas and intestines [15,16]. Therefore, for comparison, we also synthesized LW02050 (Figure 1C) by replacing the pABzA-DIG linker in LW02075 with a Pip linker to potentially facilitate excretion of the tracer via the renal pathway and reduce its uptake in normal organs/tissues. Both LW02075 and LW02050 were labeled with ^68^Ga and evaluated by biodistribution and PET imaging studies, and compared with the clinically validated [^68^Ga]Ga-SB3.

## 2. Results

### 2.1. Peptide Synthesis and Radiolabeling

LW02075 and LW02050 were obtained in 3.4% and 9.5% yields, respectively (Table S1 and Figures S1 and S2) and their corresponding nonradioactive Ga-complexed standards were obtained in 25% and 82% yields, respectively (Table S2 and Figures S3 and S4). ^68^Ga radiolabeling was conducted by microwave heating and, after HPLC purification (Table S3), the ^68^Ga-labeled tracers were obtained in 36–59% decay-corrected radiochemical yield with >114 GBq/µmol molar activity and >97% radiochemical purity.

### 2.2. Binding Affinity

**Figure 2:**
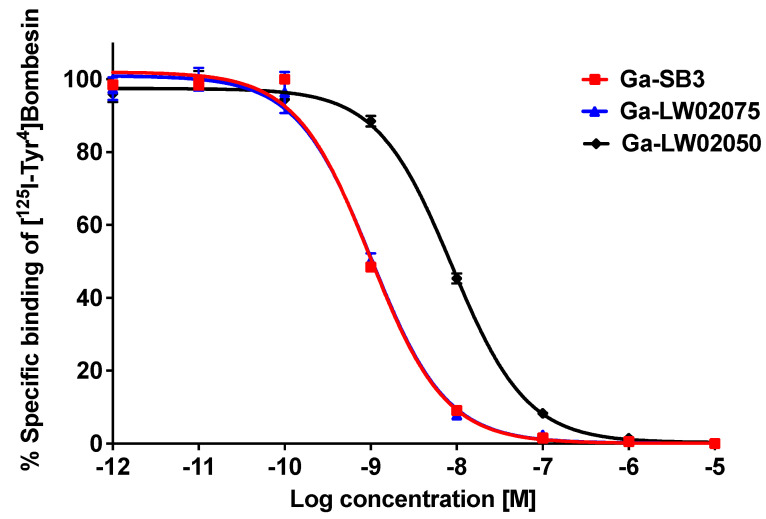
Displacement curves of [^125^I-Tyr^4^]Bombesin by Ga-LW02075, Ga-LW02050, and Ga-SB3 generated using GRPR-expressing PC-3 cells.

The K_i_ values of Ga-LW02075, Ga-LW02050, and Ga-SB3 were measured using GRPR-expressing PC-3 prostate cancer cells via a cell-based competition binding assay. Ga-SB3, Ga-LW02075, and Ga-LW02050 inhibited the binding of [^125^I-Tyr^4^]Bombesin towards PC-3 cells in a dose-dependent manner (Figure 2). The calculated K_i_ values for Ga-LW02075, Ga-LW02050, and Ga-SB3 were 1.39 ± 0.54, 8.53 ± 1.52, and 1.20 ± 0.31 nM, respectively (n = 3).

### 2.3. Antagonist Characterization and Hydrophilicity Measurement

**Figure 3:**
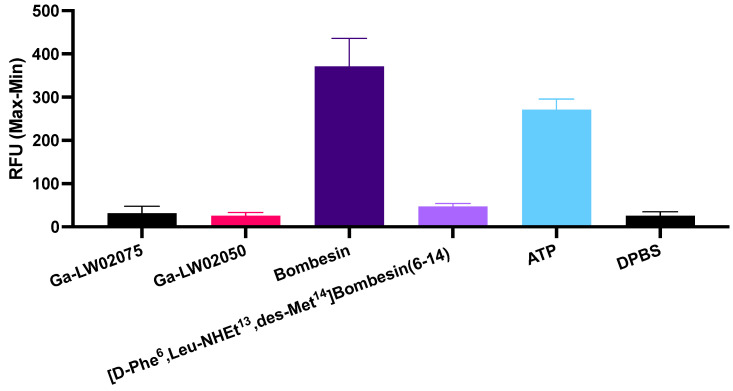
Intracellular calcium mobilization in PC-3 cells induced by Ga-LW02075, Ga-LW02050, Bombesin, [D-Phe^6^,Leu-NHEt^13^,des-Met^14^]Bombesin(6-14), ATP, and DPBS.

The antagonist characteristics of both Ga-LW02075 and Ga-LW02050 were confirmed by intracellular calcium release assay (Figure 3). ATP (50 nM, positive control) and bombesin (50 nM, agonist control) induced Ca^2+^ mobilization with 271 ± 24.4 and 371 ± 65.0 relative fluorescence units (RFUs), respectively. For 50 nM of Ga-LW02075 and Ga-LW02050, only 31.4 ± 16.5 and 25.8 ± 7.95 RFUs, respectively, were observed. These values were significantly lower than the values obtained by ATP and bombesin, and were close to those obtained using the blank control (Dulbecco’s phosphate-buffered saline, DPBS, 25.7 ± 9.24 RFUs) and the antagonist control ([D-Phe^6^,Leu-NHEt^13^,des-Met^14^]Bombesin(6-14), 50 nM, 47.2 ± 6.61 RFUs). The LogD_7.4_ values of [^68^Ga]Ga-LW02075, [^68^Ga]Ga-LW02050, and [^68^Ga]Ga-SB3 were obtained using the shake flask method, and were −2.09 ± 0.05, −2.30 ± 0.14, and −2.47 ± 0.09, respectively (n = 3).

### 2.4. PET Imaging

**Figure 4:**
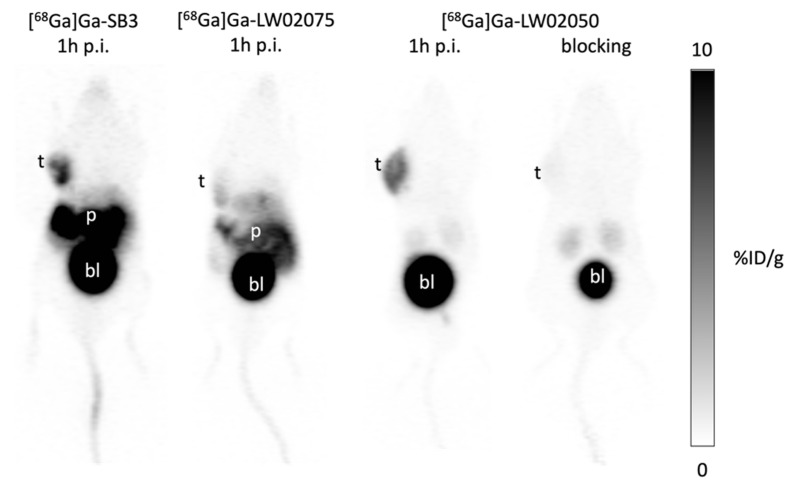
Representative PET images of [^68^Ga]Ga-SB3, [^68^Ga]Ga-LW02075, and [^68^Ga]Ga-LW02050 acquired at 1 h post injection in mice bearing PC-3 tumor xenografts. t: tumor; p: pancreas; bl: urinary bladder.

The PC-3 tumor xenografts were clearly visualized in PET images acquired at 1 h post injection using [^68^Ga]Ga-SB3 and [^68^Ga]Ga-LW02050 (Figure 4). All [^68^Ga]Ga-SB3, [^68^Ga]Ga-LW02075, and [^68^Ga]Ga-LW02050 were excreted primarily through the renal pathway, as a very high uptake in urinary bladder was observed. A high pancreas uptake was also observed by both [^68^Ga]Ga-SB3 and [^68^Ga]Ga-LW02075, while the pancreas was nearly invisible in the PET image of [^68^Ga]Ga-LW02050. Co-injection with 100 μg of nonradioactive Ga-LW02050 significantly reduced the uptake of [^68^Ga]Ga-LW02050 in the PC-3 tumor xenograft to close to the background level.

### 2.5. Ex Vivo Biodistribution

**Figure 5:**
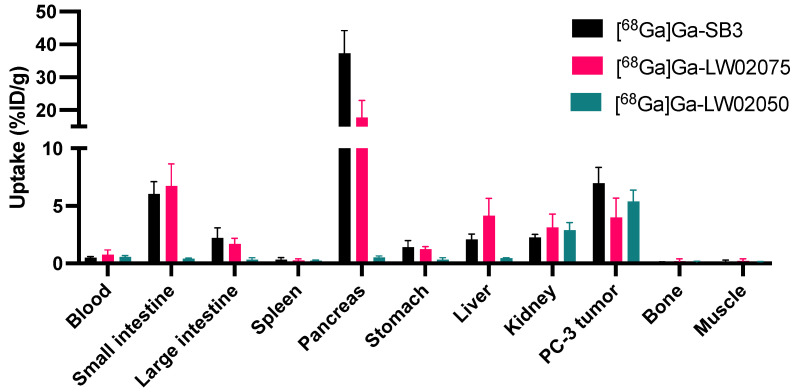
Uptake of [^68^Ga]Ga-SB3, [^68^Ga]Ga-LW02075, and [^68^Ga]Ga-LW02050 in tumors and major organs/tissues of PC-3 tumor-bearing mice at 1 h post injection.

**Figure 6:**
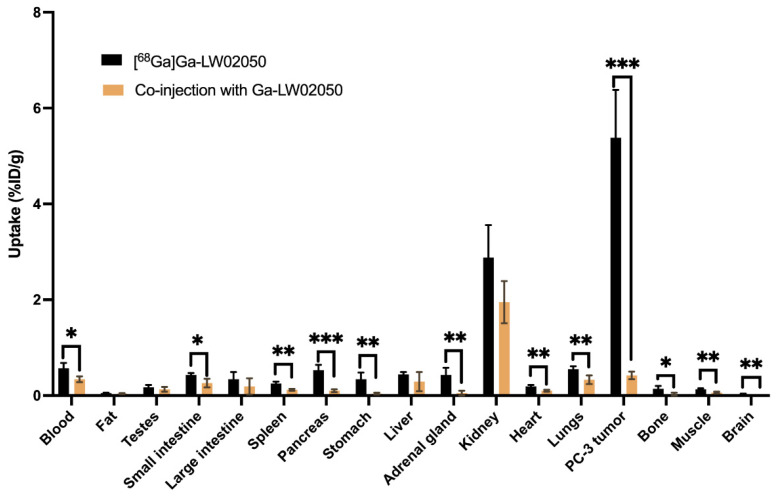
Comparison of [^68^Ga]Ga-LW02050 with/without co-injection of its nonradioactive standard (100 μg) on the uptake in PC-3 tumor xenografts and major organs/tissues in mice at 1 h post injection. * *p* < 0.05, ** *p* < 0.01, *** *p* < 0.001.

Biodistribution studies were conducted for ^68^Ga-labeled SB3, LW02075 and LW02050 in PC-3 tumor-bearing mice (n = 4). The results of the biodistribution studies are provided in Figure 5 and Figure 6 and Table S4, and are consistent with the observations from the PET images. Tumor uptake of [^68^Ga]Ga-LW02050 was comparable to that of [^68^Ga]Ga-SB3 (5.38 ± 1.00 vs. 6.98 ± 1.36 %ID/g, *p* = 0.107), while [^68^Ga]Ga-LW02075 showed a significantly lower uptake in PC-3 tumor xenografts (3.97 ± 1.71 %ID/g). Consistent with the PET images, [^68^Ga]Ga-SB3 showed the highest pancreas uptake (37.3 ± 6.90 %ID/g), which was around two-fold greater than the pancreas uptake of [^68^Ga]Ga-LW02075 (17.8 ± 5.24 %ID/g). In contrast, the pancreas uptake of [^68^Ga]Ga-LW02050 was only 0.53 ± 0.11 %ID/g. Compared to [^68^Ga]Ga-LW02050, which had a low uptake in the liver (0.44 ± 0.05 %ID/g), small intestine (0.43 ± 0.04 %ID/g), and large intestine (0.34 ± 0.15 %ID/g), both [^68^Ga]Ga-SB3 and [^68^Ga]Ga-LW02075 showed a considerably higher uptake in the liver (2.10 ± 0.45 and 4.14 ± 1.50 %ID/g, respectively), small intestine (6.03 ± 1.07 and 6.72 ± 1.94 %ID/g, respectively), and large intestine (2.20 ± 0.89 and 1.71 ± 0.47 %ID/g, respectively). The tumor-to-bone, tumor-to-muscle, tumor-to-blood, tumor-to-kidney, and tumor-to-pancreas uptake ratios of [^68^Ga]Ga-LW02050 were 43.6 ± 22.6, 44.4 ± 14.1, 9.68 ± 2.84, 1.92 ± 0.41, and 10.7 ± 4.17, respectively, significantly higher than those of [^68^Ga]Ga-LW02075 (30.6 ± 18.9, 24.8 ± 9.99, 5.51 ± 1.49, 1.28 ± 0.33, and 0.22 ± 0.05, respectively).

Consistent with the PET images, the co-injection of nonradioactive Ga-LW02050 (100 μg) reduced the average uptake of [^68^Ga]Ga-LW02050 in PC-3 tumor xenografts by 92%, from 5.38 %ID/g down to 0.42 %ID/g at 1 h post-injection (Figure 6 and Table S4). Furthermore, the average uptake of [^68^Ga]Ga-LW02050 in the pancreas was also reduced by 81% (0.53 %ID/g down to 0.10 %ID/g) by the co-injection of nonradioactive Ga-LW02050. A blocking study was also performed for [^68^Ga]Ga-LW02075. Similarly, co-injecting 100 μg of nonradioactive Ga-LW02075 reduced the average uptake of [^68^Ga]Ga-LW02075 in the PC-3 tumor xenografts by 54% (3.97 %ID/g down to 1.83 %ID/g). In addition, a significant reduction in the average pancreas uptake of [^68^Ga]Ga-LW02075 was observed. The average pancreas uptake of [^68^Ga]Ga-LW02075 declined by 91% from 17.8 %ID/g to 1.55 %ID/g (Table S4).

## 3. Discussion

Inspired by the potent GRPR antagonist sequence, D-Phe-Gln-Trp-Ala-Val-Gly-His-Leu-NHOH, reported by Devin et al., we synthesized LW02075 (Figure 1B) by replacing the *C*-terminal ethylamide in SB3 (Figure 1A) with a *C*-terminal hydroxamate. The design of LW02050 (Figure 1C), replacing the pABzA-DIG linker in LW02075 with a more hydrophilic Pip linker, was based on data from our previously reported GRPR-targeted tracers, [^68^Ga]Ga-ProBOMB1 and [^68^Ga]Ga-ProBOMB2 [15,16]. Compared to [^68^Ga]Ga-ProBOMB1 containing a pABzA-DIG linker, [^68^Ga]Ga-ProBOMB2 containing a Pip linker showed a comparable high tumor uptake but a significantly lower uptake in normal organs/tissues, practically in the pancreas. Accordingly, in this study, we also used a Pip linker instead of the pABzA-DIG linker to synthesize LW02050 for comparison.

The average K_i_(GRPR) of Ga-LW02075 was comparable with that of Ga-SB3 (1.39 ± 0.54 vs. 1.20 ± 0.31 nM, Figure 2). This indicates that replacing the *C*-terminal ethylamide in Ga-SB3 with a hydroxamate does not affect their GRPR binding affinity. In contrast, the average K_i_(GRPR) of Ga-LW02050 (8.53 ± 1.52 nM) containing the Pip linker was significantly inferior to those of Ga-SB3 and Ga-LW02075. This finding is unexpected as the Pip linker is also very popular for the design of GRPR-targeted radioligands, including the clinically validated [^68^Ga/^177^Lu]Ga/Lu-RM2 [10,11,17]. In addition, in our previous studies, we showed that the binding affinity of Ga-ProBOMB1 containing a pABzA-DIG linker was comparable to that of Ga-ProBOMB2 containing a Pip linker (K_i_(GRPR) = 3.97 ± 0.76 vs. 4.58 ± 0.67 nM) [15,16].

The antagonist characteristics of Ga-LW02075 and Ga-LW02050 were confirmed by calcium release assay because, compared with the antagonist control, [D-Phe^6^,Leu-NHEt^13^,des-Met^14^]Bombesin(6-14), there was a slightly lower calcium mobilization induced by Ga-LW02075 and Ga-LW02050 (Figure 3). The confirmed antagonist characteristics indicates that the addition of Ga-DOTA-pABzA-DIG (in LW02075) or Ga-DOTA-Pip (in LW02050) to the *N*-terminus of the reported GRPR antagonist sequence, D-Phe-Gln-Trp-Ala-Val-Gly-His-Leu-NHOH, does not change its agonist/antagonist characteristics.

The hydrophilic nature of both [^68^Ga]Ga-LW02075 and [^68^Ga]Ga-LW02050 was verified by their low LogD_7.4_ values of −2.09 ± 0.05 and −2.30 ± 0.14, respectively. Compared to [^68^Ga]Ga-SB3 (LogD_7.4_ = −2.47 ± 0.09), [^68^Ga]Ga-LW02075 is slightly more lipophilic (*p* = 0.00043). This indicates that replacing the *C*-terminal ethylamide of [^68^Ga]Ga-SB3 with a hydroxamate led to a less hydrophilic [^68^Ga]Ga-LW02075. In contrast, replacing both the *C*-terminal ethylamide and pABzA-DIG linker in [^68^Ga]Ga-SB3 with a hydroxamate and a Pip linker, respectively, led to [^68^Ga]Ga-LW02050 with a comparable hydrophilicity (LogD_7.4_ = −2.47 ± 0.09 vs. −2.30 ± 0.14, *p* = 0.112). This indicates that replacing the pABzA-DIG linker with a cationic Pip linker can increase the radioligands’ hydrophilicity.

The observations from PET images were consistent with the obtained biodistribution data (Figure 4 and Figure 5 and Table S4). A good in vivo GRPR-targeting capability of [^68^Ga]Ga-SB3 and [^68^Ga]Ga-LW02050 was confirmed as PC-3 tumor xenografts were clearly visualized in the PET images, while [^68^Ga]Ga-LW02075 showed a lower uptake in PC-3 tumor xenografts. Although the GRPR binding affinity of Ga-LW02075 was significantly better than that of Ga-LW02050 (K_i_ = 1.39 ± 0.54 vs. 8.53 ± 1.52 nM, *p* = 0.0016), [^68^Ga]Ga-LW02075 showed a lower tumor uptake (3.97 ± 1.71 vs. 5.38 ± 1.00 %ID/g). One possible explanation for these observations is the better hydrophilicity of [^68^Ga]Ga-LW02050 resulting from the incorporation of the more hydrophilic cationic Pip linker. All [^68^Ga]Ga-SB3, [^68^Ga]Ga-LW02075, and [^68^Ga]Ga-LW02050 were excreted primarily through the renal pathway with extremely high radioactivity accumulated in the urinary bladders. as observed from the PET images. A moderate hepatobiliary excretion of [^68^Ga]Ga-SB3 and [^68^Ga]Ga-LW02075 was also observed, as both tracers showed higher uptake values in the liver, small intestine, and large intestine when compared with those of [^68^Ga]Ga-LW02050. This is likely due to the incorporation of the cationic Pip linker in [^68^Ga]Ga-LW02050, leading to a lower extent of hepatobiliary excretion. The pancreas was also clearly visualized in the PET images of [^68^Ga]Ga-SB3 and [^68^Ga]Ga-LW02075, but not in the PET image of [^68^Ga]Ga-LW02050. This was also confirmed by the obtained biodistribution data: [^68^Ga]Ga-SB3 showed the highest pancreas uptake (37.3 ± 6.90 %ID/g) followed by [^68^Ga]Ga-LW02075 (17.8 ± 5.24 %ID/g), while the uptake of [^68^Ga]Ga-LW02050 in the pancreas was only 0.53 ± 0.11 %ID/g. The minimal uptake of [^68^Ga]Ga-LW02050 in the liver, intestine, and pancreas might also contribute to the higher uptake in PC-3 tumor xenografts compared to that of [^68^Ga]Ga-LW02075.

The good tumor uptake of [^68^Ga]Ga-LW02050 and its minimal uptake in normal organs/tissues result in excellent tumor-to-background imaging contrast (Figure 4). Our data support [^68^Ga]Ga-LW02050 as a promising PET tracer for detecting GRPR-expressing lesions, even for those adjacent to or in the pancreas. In addition, our findings suggest that LW02050 is a promising pharmacophore for the design of GRPR-targeted radiopharmaceuticals, especially when labeled with α- or β-emitters for therapeutic application to minimize toxicity to the pancreas.

Blocking studies were performed for both [^68^Ga]Ga-LW02075 and [^68^Ga]Ga-LW02050 by the co-injection with 100 μg of their respective nonradioactive standard (Figure 4 and Figure 6, and Table S4). Consistent with the observation from the PET image, the tumor uptake of [^68^Ga]Ga-LW02050 reduced by 92%, confirming its specific uptake in PC-3 tumor xenografts. The significant reduction in [^68^Ga]Ga-LW02050 in the pancreas (by 81%) is in agreement with the fact that the pancreas is the highest GRPR-expressing normal organ [1,2,3]. For [^68^Ga]Ga-LW02075, there was 54% reduction in PC-3 tumor uptake and 91% reduction in pancreas uptake by the co-injection of 100 μg of nonradioactive Ga-LW02075. This suggests that [^68^Ga]Ga-LW02075 might have a higher binding affinity or be more selective toward the mouse GRPR expressed by the mouse pancreas than the human GRPR expressed by the PC-3 tumor xenografts.

## 4. Materials and Methods

### 4.1. General Methods

SB3 and its nonradioactive Ga-complexed standard were synthesized following previously reported procedures [18]. All other reagents and solvents were purchased from commercial sources. LW02075 and LW02050 were synthesized on solid phase using an AAPPTec (Louisville, KY, USA) Endeavor 90 peptide synthesizer. Agilent (Santa Clara, CA, USA) HPLC systems equipped with a model 1200 quaternary pump, a model 1200 UV absorbance detector (220 nm), and a Bioscan (Washington, DC, USA) NaI scintillation detector were used for the purification and quality control of LW02075, LW02050, and their ^nat^Ga/^68^Ga-complexed analogs. HPLC purification was performed using a semi-preparative column (Luna C18, 5 µ, 250 × 10 mm), and quality control was conducted with an analytical column (Luna C18, 5 µ, 250 × 4.6 mm) purchased from Phenomenex (Torrance, CA, USA). A Labconco (Kansas City, MO, USA) FreeZone 4.5 Plus freeze-drier was used for lyophilizing the collected HPLC eluates. An AB SCIEX (Framingham, MA, USA) 4000 QTRAP mass spectrometer system with an ESI ion source was used for MS analysis. ^68^Ga was eluted from an ITM Medical Isotopes GmbH (Munich, Germany) generator and purified via a DGA resin column from Eichrom Technologies LLC (Lisle, IL, USA). C18 Sep-Pak cartridges (1 cm^3^, 50 mg) were purchased from Waters (Milford, MA, USA). The radioactivity of ^68^Ga-labeled peptides was measured via a Capintec (Ramsey, NJ, USA) CRC^®^-25R/W dose calibrator. The radioactivity values of the biodistribution samples were counted on a Perkin Elmer (Waltham, MA, USA) Wizard2 2480 automatic gamma counter.

### 4.2. Synthesis of DOTA-Conjugated Precursors

The synthesis of LW02075 and LW02050 was conducted using Fmoc peptide chemistry on solid phase. Fmoc-hydroxylamine-2-Cl-Trt resin (0.1 mmol) was treated with 20% piperidine in *N*,*N*-dimethylformamide (DMF) to remove the Fmoc protecting group. After removing the Fmoc protecting group, Fmoc-protected amino acids (5 eq.) were pre-activated with HATU (5 eq.), HOAt (5 eq.), and *N*,*N*-diisopropylethylamine (DIEA, 15 eq.), before being sequentially coupled to the resin. Pre-activated with HATU (5 eq.), HOAt (5 eq.), and DIEA (15 eq.), Fmoc-*p*-aminomethylaniline-diglycolic acid (Fmoc-pABzA-DIG, 5 eq.) linker, and Fmoc-4-amino-(1-carboxymethyl)piperidine (Fmoc-Pip-OH linker, 5 eq.) linker were coupled to the resins for LW02075 and LW02050, respectively. At the end, DOTA(*t*Bu)_3_ (5 eq.) pre-activated with HATU (5 eq.) and DIEA (25 eq.) was coupled to the *N*-terminus.

The peptides were cleaved from the resin at room temperature for 4 h using a mixture of trifluoroacetic acid (TFA, 81.5%), triisopropylsilane (TIS 1.0%), water (5%), 2,2′-(ethylenedioxy)diethanethiol (DODT, 2.5%), thioanisole (5%), and phenol (5%). The cleaved peptides were filtrated and precipitated by the addition of cold diethyl ether. The crude peptides were collected after a 10 min centrifugation at 1000× *g*, and then purified by HPLC. The eluates containing the desired products were collected and then lyophilized. The HPLC conditions, retention times, isolated yields, purities, and MS confirmations of LW02075 and LW02050 are provided in Table S1 and Figures S1 and S2.

### 4.3. Synthesis of Nonradioactive Ga-Complexed Standards

The nonradioactive Ga-complexed standards of LW02075 and LW02050 were prepared by incubating the DOTA-conjugated precursor (1 eq.) with GaCl_3_ (5 eq.) in NaOAc buffer (pH 4.5, 0.1 M, 500 µL) at 80 °C for 15 min. The reaction mixture was then purified via HPLC after cooling down to room temperature. The collected HPLC eluates containing the desired nonradioactive Ga-complexed standard were lyophilized via a Labconco (Kansas City, MO, USA) FreeZone 4.5 Plus freeze-drier. The HPLC conditions, retention times, isolated yields, purities, and MS confirmations of Ga-LW02075 and Ga-LW02050 are provided in Table S2 and Figures S3 and S4.

### 4.4. Synthesis of ^68^Ga-Labeled Tracers

The Ga-68 radiolabeling experiments were conducted following procedures reported in the literature [15,16]. Purified ^68^GaCl_3_ in 0.5 mL water was added into a G4 glass vial preloaded with 0.7 mL of HEPES buffer (2 M, pH 5.0) and 10 μL of the precursor solution (1 mM). The radiolabeling reaction was performed with 1 min microwave heating in a Monowave200 (Anton Paar, Graz, Austria) microwave reactor at 100 °C, followed by HPLC purification. The eluate fraction containing the desired radiolabeled product was collected and diluted with 50 mL of water. The diluted product solution was passed through a C18 Sep-Pak cartridge which was pre-washed with ethanol (1 mL) and water (2 mL). The ^68^Ga-labeled product was eluted with ethanol (0.4 mL) and diluted with DPBS, which contained 0.1% ascorbic acid for both PET imaging and biodistribution studies. Quality control was performed on HPLC using the analytical column. The HPLC conditions and retention times are listed in Table S3.

### 4.5. LogD_7.4_ Measurement

LogD_7.4_ values of [^68^Ga]Ga-LW02075, [^68^Ga]Ga-LW02050, and [^68^Ga]Ga-SB3 were measured via the shake flask method. An aliquot of the ^68^Ga-labeled tracer (~1.85 MBq) was added to a 15 mL falcon tube containing 3 mL of n-octanol and 3 mL of DPBS (pH 7.4) followed by 1 min of vortex (n = 3). After that, the mixture was centrifuged at 5000 rpm for 10 min, and samples of the n-octanol (1 mL) and buffer (1 mL) layers were collected and counted on a Perkin Elmer (Waltham, MA, USA) Wizard2 2480 automatic gamma counter. The LogD_7.4_ values of [^68^Ga]Ga-LW02075, [^68^Ga]Ga-LW02050, and [^68^Ga]Ga-SB3 were calculated following the equation: LogD_7.4_ = log_10_[(counts in the n-octanol phase)/(counts in the buffer phase)].

### 4.6. Cell Culture

Human PC-3 prostate adenocarcinoma cells were obtained from ATCC (via Cedarlane, Burlington, ON, Canada) and cultured in RPMI 1640 medium (Life Technologies Corporations, Carlsbad, CA, USA) containing 10% fetal bovine serum (FBS), penicillin (100 U/mL), and streptomycin (100 μg/mL) at 37 °C in an MCO-19AIC humidified incubator (Panasonic Healthcare, Tokyo, Japan) with 5% CO_2_. An IMPACT Rodent Pathogen Test (IDEXX BioAnalytics, Columbia, MO, USA) was performed to confirm that the cells were pathogen-free. Cells grown to 80–90% confluence were harvested after 1 min trypsinization. The cell concentration was counted via a Moxi mini automated cell counter (ORFLO Technologies, Ketchum, ID, USA).

### 4.7. Fluorometric Calcium Release Assay

The antagonist/agonist characteristics of Ga-LW02075 and Ga-LW02050 were determined following previously published procedures [15,16]. A 96-well clear-bottomed black plate was seeded with 5 × 10^4^ PC-3 cells/well in 100 μL growth media 24 h prior to the assay. A calcium-sensitive dye (FLIPR Calcium 6 assay kit from Molecular Devices, San Jose, CA, USA) dissolved in the loading buffer (100 μL/well) was added into the plate followed by 1 h incubation at 37 °C. Then, the plate was transferred into a FlexStation 3 microplate reader (Molecular Devices, San Jose, CA, USA) to record the fluorescent signals. Ga-complexed SB3 derivatives (50 nM), [D-Phe^6^,Leu-NHEt^13^,des-Met^14^]Bombesin(6-14) (50 nM, antagonist control), bombesin (50 nM, agonist control), adenosine triphosphate (ATP, 50 nM, positive control), or DPBS (blank control) were added. The fluorescent signals were acquired for 2 min (λ_Ex_ = 485 nm; λ_Em_ = 525 nm; n = 3). The relative fluorescent units (RFUs = max − min) were calculated to determine the agonistic/antagonistic characteristics of the tested ligands.

### 4.8. In Vitro Competition Binding Assay

Inhibition constants (K_i_) of Ga-SB3, Ga-LW02075, and Ga-LW02050 toward GRPR were measured by in vitro competition binding assay following previously published procedures [16,19,20]. The assays were conducted in triplicate with varied concentrations (10 μM to 1 pM) of Ga-SB3, Ga-LW02075, and Ga-LW02050. Briefly, 2 × 10^5^ cells/well of PC-3 cells were seeded in 24-well poly-D-lysine plates 24 h prior to the assay. The growth medium was replaced with 400 μL of reaction medium (RPMI 1640 containing 2 mg/mL bovine serum albumin (BSA) and 20 mM HEPES), followed by 1 h incubation at 37 °C. The tested ligands in 50 μL reaction medium and 0.01 nM [^125^I-Tyr^4^]Bombesin in 50 μL reaction medium were added into the wells, and then incubated at 37 °C with moderate agitation for 1 h. PC-3 cells were harvested by trypsinization after two rounds of DPBS washing, and counted on a Perkin Elmer (Waltham, MA, USA) Wizard2 2480 automatic gamma counter. Data analysis was performed using nonlinear regression with GraphPad (San Diego, CA, USA) Prism 10 software (Version 10.1.1).

### 4.9. Ex Vivo Biodistribution and PET/CT Imaging

Imaging and biodistribution studies were performed on male NOD.Cg-Rag1^tm1Mom^ Il2rg^tm1Wjl^/SzJ (NRG) mice following previously published procedures [21]. The experiments were conducted following the guidelines established by the Canadian Council on Animal Care and approved by Animal Ethics Committee of the University of British Columbia. The mice were sedated (2.5% isoflurane in oxygen) and injected with 5 × 10^6^ PC-3 cells (100 µL; 1:1 PBS/Matrigel) subcutaneously behind the left shoulder. The tumor-bearing mice were used for PET/CT imaging and biodistribution studies when the tumor grew to 5–8 mm in diameter.

PET/CT imaging experiments were performed on a Siemens (Knoxville, TN, USA) Inveon micro PET/CT scanner. Each tumor-bearing mouse was injected with ~3–6 MBq of ^68^Ga-labeled tracer through a lateral caudal tail vein under anaesthesia. At around 50 min post injection, a 10 min CT scan was obtained first for localization and attenuation correction after segmentation for reconstructing the PET image, followed by a 10 min static PET scan.

For biodistribution studies, the mice were injected with the radiotracer (~2–4 MBq), as described above. Blocking of [^68^Ga]Ga-LW02075 and [^68^Ga]Ga-LW02050 was conducted by co-injection with 100 μg nonradioactive Ga-LW02075 and Ga-LW02050, respectively, through a lateral caudal tail vein under anaesthesia. At 1 h post injection, the mice were sedated and euthanized by CO_2_ inhalation. Blood was collected via cardiac puncture, and organs/tissues of interest were collected, weighed, and counted on a Perkin Elmer (Waltham, MA, USA) Wizard2 2480 automatic gamma counter.

### 4.10. Statistical Analysis

Statistical analyses were conducted with Student’s *t*-test using the Microsoft (Redmond, WA, USA) Excel software (Version 16.84) for the data obtained from biodistribution studies. Comparison of the biodistribution data of [^68^Ga]Ga-LW02050 and [^68^Ga]Ga-SB3 was conducted with unpaired two-tailed test, and the comparison of the biodistribution data with/without co-injection of the nonradioactive standard was performed by unpaired one-tailed test. Statistically significant differences were considered present when the adjusted *p* value was <0.05.

## 5. Conclusions

In this study, we report the first *C*-terminal hydroxamate-derived GRPR-targeted radioligands based on the reported potent GRPR antagonist, D-Phe-Gln-Trp-Ala-Val-Gly-His-Leu-NHOH. We demonstrated that the addition of Ga-DOTA-pABzA-DIG (in Ga-LW02075) or Ga-DOTA-Pip (in Ga-LW02050) to the *N*-terminus of D-Phe-Gln-Trp-Ala-Val-Gly-His-Leu-NHOH retains its high GRPR binding affinity. Compared with the closely related and clinically validated [^68^Ga]Ga-SB3, [^68^Ga]Ga-LW02050 showed comparable tumor uptake but a much less hepatobiliary excretion and especially a dramatically lower uptake in the pancreas. Our data suggest that [^68^Ga]Ga-LW02050 is a promising PET tracer for detecting GRPR-expressing lesions, even for lesions adjacent to or in the pancreas. Due to the minimal pancreas uptake of [^68^Ga]Ga-LW02050, LW02050 is also promising for the radioligand therapy application when labeled with a α- or β-emitters to minimize toxicity to the pancreas.

## 6. Patents

The compounds disclosed in this report are covered by a recent US provisional patent application (application number: 63/538,185; filing date: 13 September 2023). Kuo-Shyan Lin, François Bénard, Lei Wang, and Chao-Cheng Chen are listed as inventors in this filed provisional patent application.

## Data Availability

The data presented in this study are available in the Supplementary Materials.

## Supplementary Materials

The following supporting information can be downloaded at: https://www.mdpi.com/article/10.3390/molecules29133102/s1, Table S1: HPLC purification conditions and MS characterizations of LW02075 and LW02050; Figure S1: The MS spectrum of LW02075; Figure S2: The MS spectrum of LW02050; Table S2: HPLC purification conditions and MS characterizations of Ga-LW02075 and Ga-LW02050; Figure S3: The MS spectrum of Ga-LW02075; Figure S4: The MS spectrum of Ga-LW02050; Table S3: HPLC conditions for the purification and quality control of [^68^Ga]Ga-LW02075, [^68^Ga]Ga-LW02050, and [^68^Ga]Ga-SB3; Table S4: Biodistribution and uptake ratios of ^68^Ga-labeled GRPR-targeted tracers in PC-3 tumor-bearing mice.
