# Supplementary material for: Synthesis and Evaluation of the First 68Ga-Labeled C-Terminal Hydroxamate-Derived Gastrin-Releasing Peptide Receptor-Targeted Tracers for Cancer Imaging with Positron Emission Tomography
Source: Molecules. 2024 Jun 28;29(13):3102. doi: 10.3390/molecules29133102 (PMC11243334; doi:10.3390/molecules29133102)
Supplement: Supplementary file 1 [file molecules-29-03102-s001.zip › molecules-3069374-supplementary.pdf]

SUPPLEMENTAL INFORMATION

Table S1: HPLC purification conditions and MS characterizations of LW02075 and LW02050.

Compound name	HPLC conditions	Retention time (min)	Yield (%)	Calculated mass (m/z)	Found (m/z)	Purity
LW02075	22% CH_3CN and 0.1% TFA in H_2O	28.6	3.4	$[M+2H]^{2+}$ 789.9	$[M+2H]^{2+}$ 790.1	>99%
LW02050	20% CH_3CN and 0.1% TFA in H_2O	15.8	9.5	$[M+2H]^{2+}$ 749.9	$[M+2H]^{2+}$ 750.0	>99%

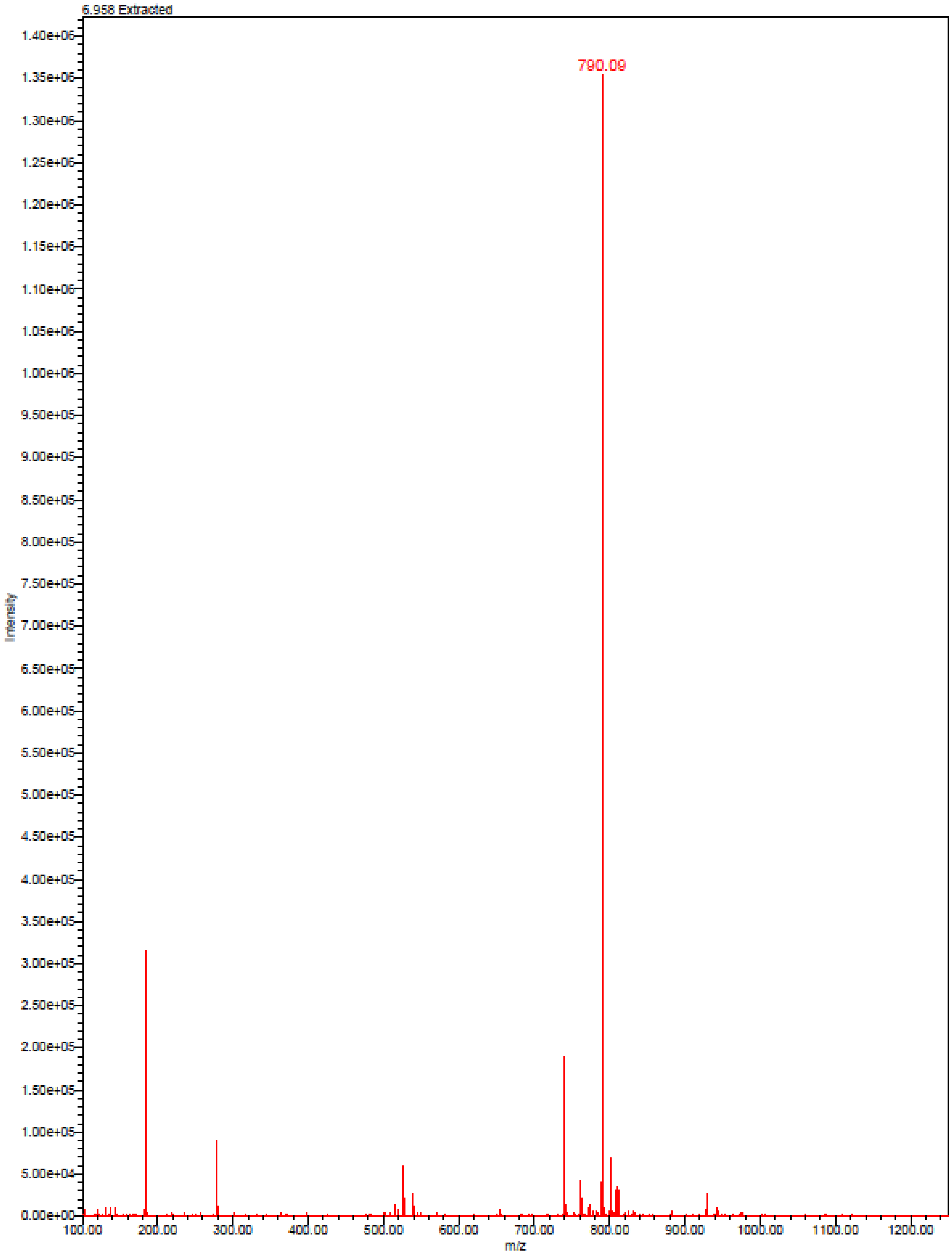

Figure S1: The MS spectrum of LW02075: calculated $[M+2H]^{2+}$ (m/z) 789.9; found 790.1.

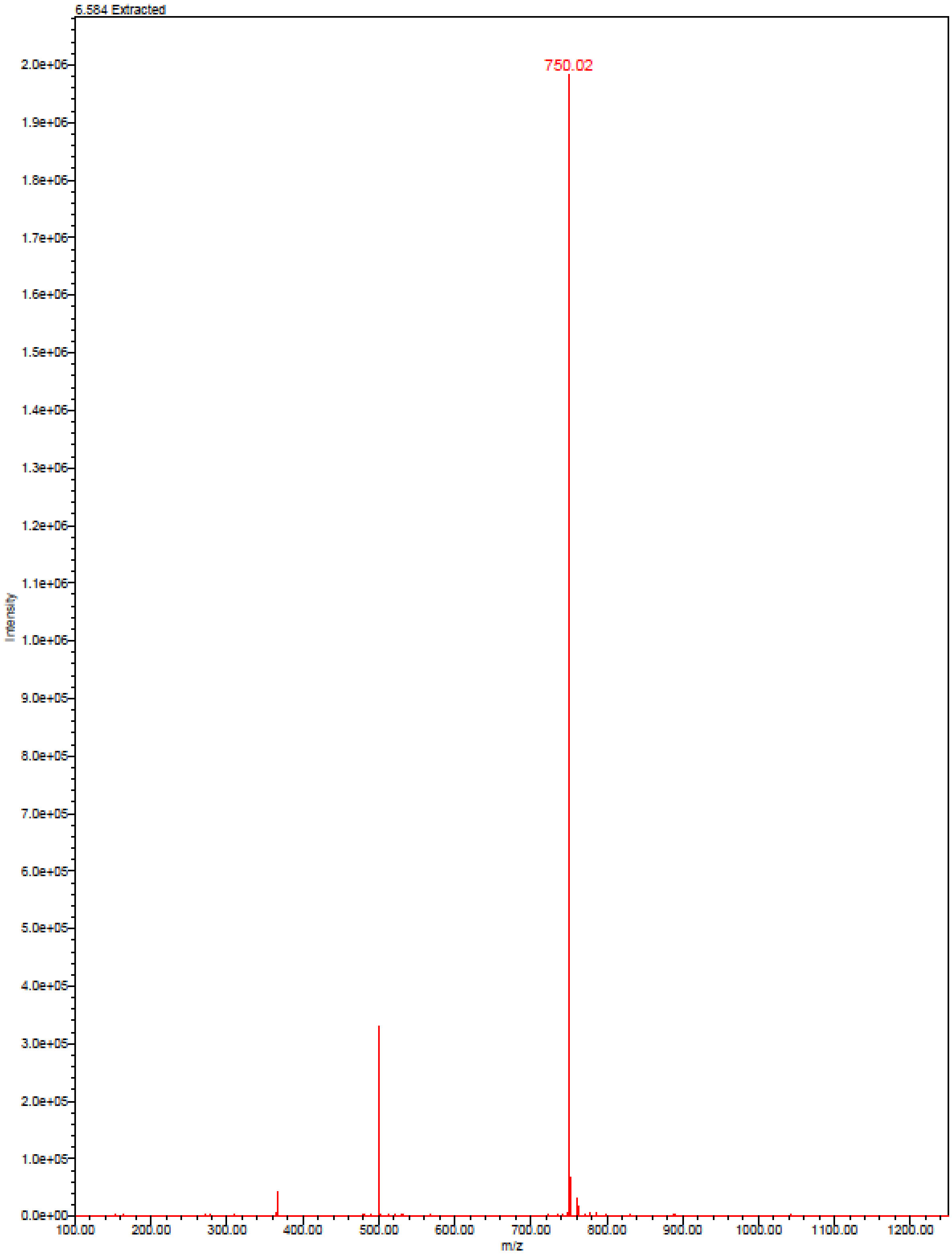

Figure S2: The MS spectrum of LW02050: calculated $[M+2H]^{2+}$ (m/z) 749.9; found 750.0.

Table S2: HPLC purification conditions and MS characterizations of Ga-LW02075 and Ga-LW02050.

Compound name	HPLC conditions	Retention time (min)	Yield (%)	Calculated mass (m/z)	Found (m/z)	Purity
Ga-LW02075	25% CH_3CN and 0.1% TFA in H_2O	17.3	25	$[M+2H]^{2+}$ 822.9	$[M+2H]^{2+}$ 823.5	>99%
Ga-LW02050	20% CH_3CN and 0.1% TFA in H_2O	22.0	82	$[M+2H]^{2+}$ 782.9	$[M+2H]^{2+}$ 783.2	>99%

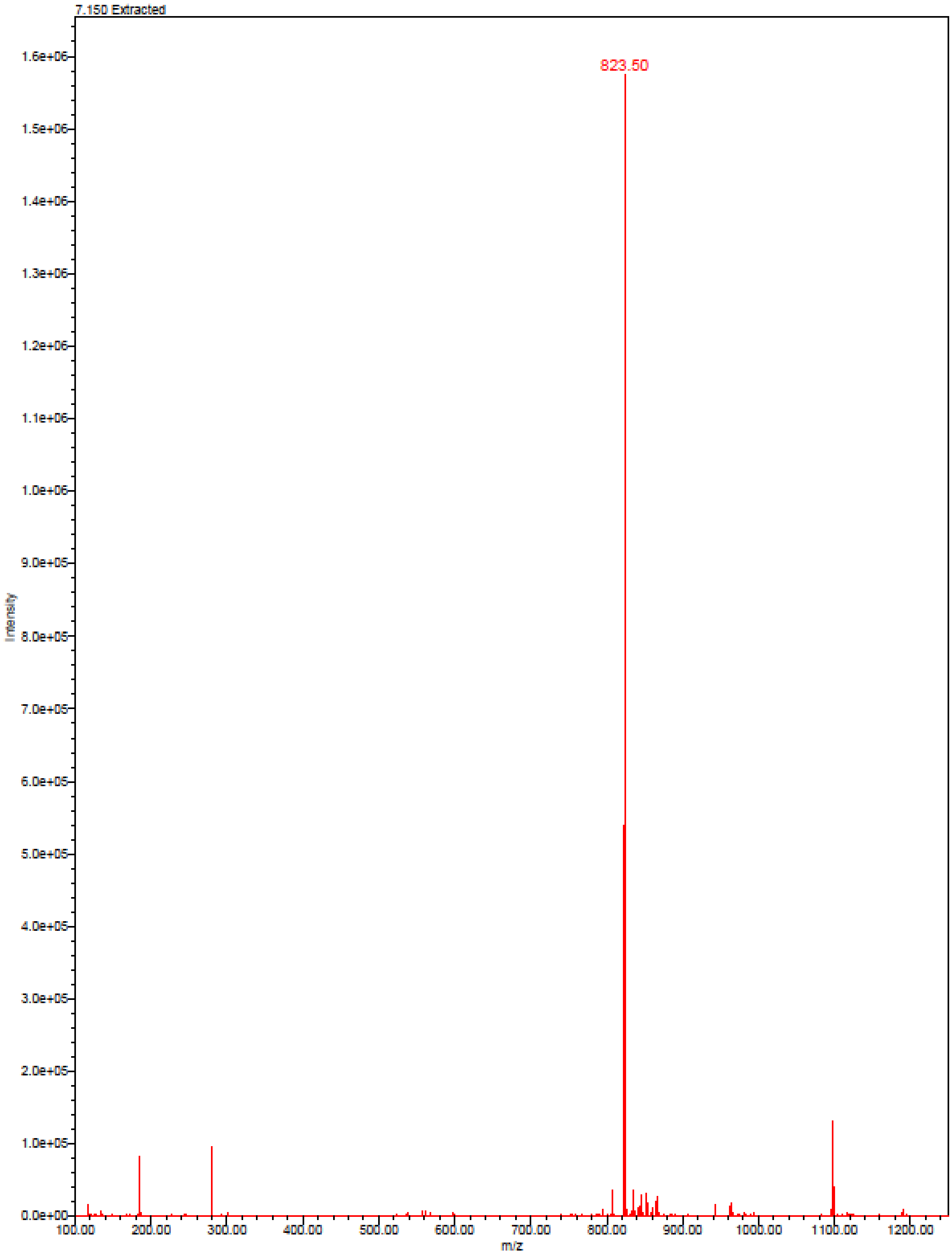

Figure S3: The MS spectrum of Ga-LW02075: calculated $[M+2H]^{2+}$ (m/z) 822.9; found 823.5.

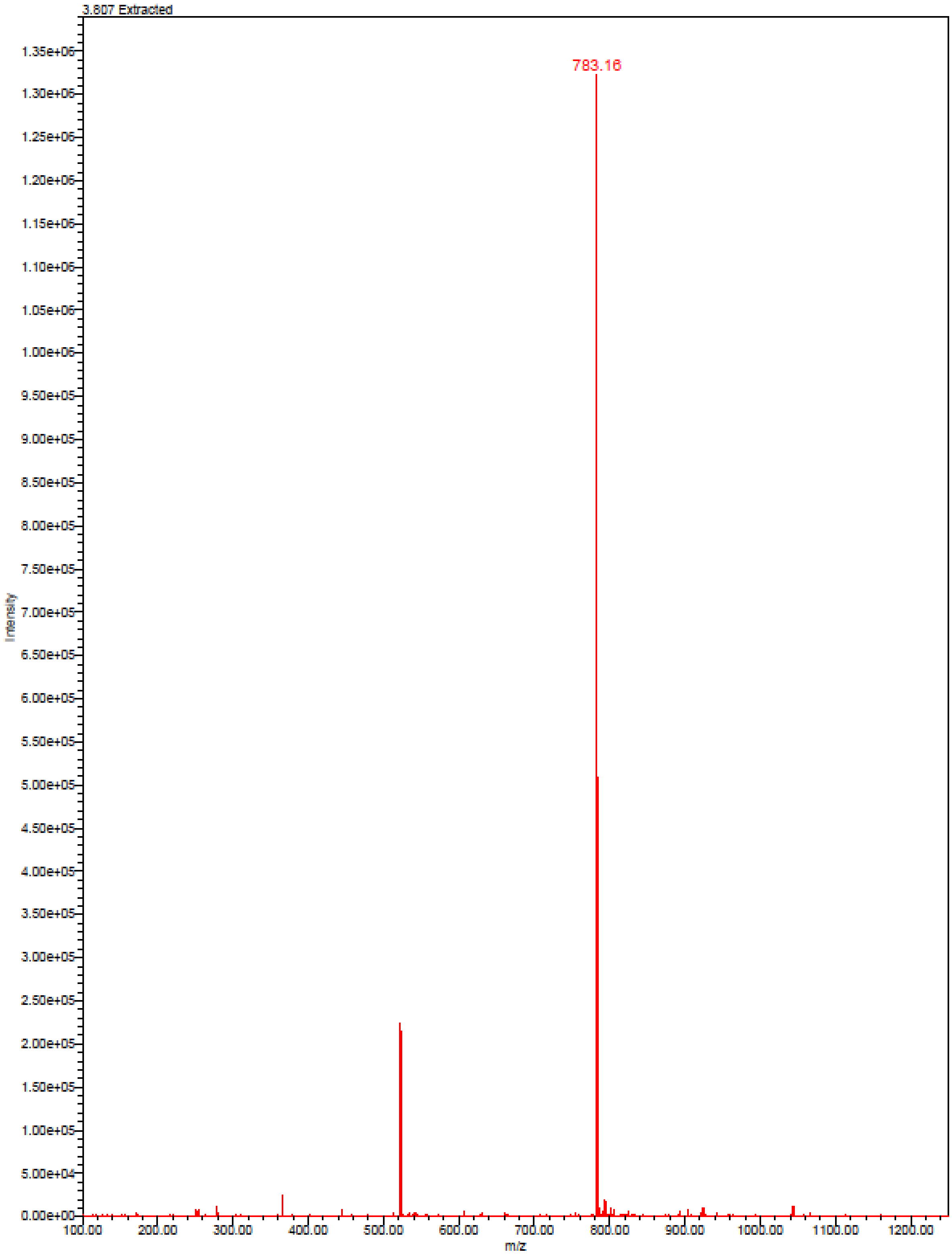

Figure S4: The MS spectrum of Ga-LW02050: calculated $[M+2H]^{2+}$ (m/z) 782.9; found 783.2.

Table S3: HPLC conditions for the purification and quality control of [^{68}Ga]Ga-LW02075, [^{68}Ga]Ga-LW02050, and [^{68}Ga]Ga-SB3. FA: formic acid.

Compound name	HPLC conditions		Retention time (min)
[^{68}Ga]Ga-LW02075	Semi-Prep	20% CH_3CN and 0.1% FA in H_2O; flow rate 4.5 mL/min	17.0
	QC	21% CH_3CN and 0.1% FA in H_2O; flow rate 2.0 mL/min	7.7
[^{68}Ga]Ga-LW02050	Semi-Prep	15% CH_3CN and 0.1% FA in H_2O; flow rate 4.5 mL/min	15.8
	QC	18% CH_3CN and 0.1% FA in H_2O; flow rate 2 mL/min	5.5
[^{68}Ga]Ga-SB3	Semi-Prep	29% CH_3CN and 0.1% FA in H_2O; flow rate 4.5 mL/min	22.5
	QC	32% CH_3CN and 0.1% FA in H_2O; flow rate 2 mL/min	11.0

Table S4: Biodistribution (mean ± SD, n = 4) and uptake ratios of ^{68}Ga-labeled GRPR-targeted tracers in PC-3 tumor-bearing mice. The mice in the blocked group were co-injected with 100 μg of their nonradioactive Ga-complexed standard (Ga-LW02075 or Ga-LW02050). Statistical analyses were conducted to compare uptake values and uptake ratios between baseline and blocked groups. * $p < 0.05$, ** $p < 0.01$, *** $p < 0.001$.

Tissue	[^{68}Ga]Ga-SB3	[^{68}Ga]Ga-LW02075		[^{68}Ga]Ga-LW02050	
(%ID/g)	1 h	1 h	1 h blocked	1 h	1 h blocked
Blood	0.50 ± 0.09	0.76 ± 0.41	1.15 ± 0.26	0.57 ± 0.11	0.34 ± 0.06*
Fat	0.05 ± 0.01	0.08 ± 0.05	0.12 ± 0.02	0.05 ± 0.01	0.04 ± 0.01
Testes	0.13 ± 0.01	0.17 ± 0.10	0.30 ± 0.06	0.17 ± 0.05	0.13 ± 0.05
Small intestine	6.03 ± 1.07	6.72 ± 1.94	5.62 ± 1.86	0.43 ± 0.04	0.26 ± 0.09*
Large intestine	2.20 ± 0.89	1.71 ± 0.47	0.40 ± 0.17*	0.34 ± 0.15	0.19 ± 0.17
Spleen	0.33 ± 0.17	0.26 ± 0.13	0.35 ± 0.12	0.25 ± 0.04	0.12 ± 0.02**
**Pancreas**	**37.3 ± 6.90**	**17.8 ± 5.24**	**1.55 ± 0.83*****	**0.53 ± 0.11**	**0.10 ± 0.03*****
Stomach	1.42 ± 0.57	1.23 ± 0.22	0.73 ± 0.29*	0.34 ± 0.14	0.04 ± 0.02**
Liver	2.10 ± 0.45	4.14 ± 1.50	5.92 ± 1.69	0.44 ± 0.05	0.29 ± 0.20
Adrenal glands	2.07 ± 0.66	4.42 ± 2.41	1.02 ± 0.59*	0.43 ± 0.15	0.05 ± 0.05**
Kidneys	2.26 ± 0.26	3.11 ± 1.18	4.98 ± 1.59	2.88 ± 0.68	1.95 ± 0.44
Heart	0.16 ± 0.02	0.24 ± 0.13	0.34 ± 0.04	0.19 ± 0.03	0.10 ± 0.02**
Lungs	1.18 ± 0.23	0.75 ± 0.41	0.94 ± 0.17	0.55 ± 0.06	0.33 ± 0.09**
**PC-3 tumor**	**6.98 ± 1.36**	**3.97 ± 1.71**	**1.83 ± 0.22***	**5.38 ± 1.00**	**0.42 ± 0.08*****
Bone	0.12 ± 0.01	0.20 ± 0.20	0.21 ± 0.07	0.14 ± 0.06	0.03 ± 0.03*
Muscle	0.18 ± 0.10	0.21 ± 0.18	0.27 ± 0.14	0.13 ± 0.02	0.06 ± 0.02**
Brain	0.04 ± 0.01	0.03 ± 0.01	0.03 ± 0.01	0.03 ± 0.01	0.01 ± 0.00**
Tumor/bone	61.0 ± 15.5	30.6 ± 18.9	9.30 ± 2.39	43.6 ± 22.6	27.1 ± 18.1
Tumor/muscle	48.2 ± 24.9	24.8 ± 9.99	7.85 ± 3.13*	44.4 ± 14.1	8.28 ± 2.70**
Tumor/blood	14.4 ± 3.88	5.51 ± 1.49	1.65 ± 0.40**	9.68 ± 2.84	1.23 ± 0.14**
Tumor/kidney	3.09 ± 0.51	1.28 ± 0.33	0.39 ± 0.09**	1.92 ± 0.41	0.22 ± 0.05***
Tumor/pancreas	0.19 ± 0.03	0.22 ± 0.05	1.46 ± 0.76*	10.7 ± 4.17	4.30 ± 0.83*
